# Simultaneous Totally Laparoscopic Distal Gastrectomy and Anterior Resection for Synchronous Gastric and Colon Cancer

**DOI:** 10.7759/cureus.15692

**Published:** 2021-06-16

**Authors:** Beslen Goksoy

**Affiliations:** 1 General Surgery, Sehit Prof. Dr. Ilhan Varank Sancaktepe Training and Research Hospital, University of Health Sciences, İstanbul, TUR

**Keywords:** synchronous, gastric cancer, colon cancer, laparoscopic distal gastrectomy, laparoscopic anterior resection

## Abstract

Although simultaneous open surgery for synchronous gastric and colon cancer has been reported frequently to date, total laparoscopic resection has been documented rarely. A 63-year-old male patient who presented with complaints of abdominal pain and constipation was diagnosed with synchronous gastric and sigmoid colon cancer. Simultaneous total laparoscopic distal gastrectomy (Roux-en-Y anastomosis and D2 lymph node dissection) and anterior resection were performed with a total of five ports. Total operation time was 310 min. and estimated blood loss was 175 mL. Histopathological examination result was reported as well-differentiated adenocarcinoma for the stomach and moderately differentiated adenocarcinoma for the colon. Staging result was Stage IIA (pT3N0M0, American Joint Committee on Cancer (AJCC) 8th Edition) for both cancers. The patient received postoperative adjuvant chemotherapy. He remains under follow-up at 21 months without any recurrence. With the improved techniques and increased experience in minimally invasive surgery, combined laparoscopic curative resection can be safely performed for gastric and colon cancer.

## Introduction

According to Global Cancer Statistics 2020 (GLOBOCAN 2020) data, colorectal cancer ranks third and gastric cancer ranks fifth in the incidence of solid organ cancers worldwide [1]. The incidence of synchronous cancer in addition to gastric cancer is a rare condition seen at a rate of 2.5% to 3.4%, where the most common another primary cancer is colorectal cancer (20.7%) followed by lung (12%), and liver (11%) [2,3]. Although simultaneous open and laparoscopic-assisted resections have been previously reported for both cancers, the curative treatment of which is surgery [4-6], total laparoscopic resection is rather rare. In this study, we present a case who underwent simultaneous total laparoscopic curative resection for synchronous gastric and sigmoid colon cancer.

## Case presentation

**Figure 1:**
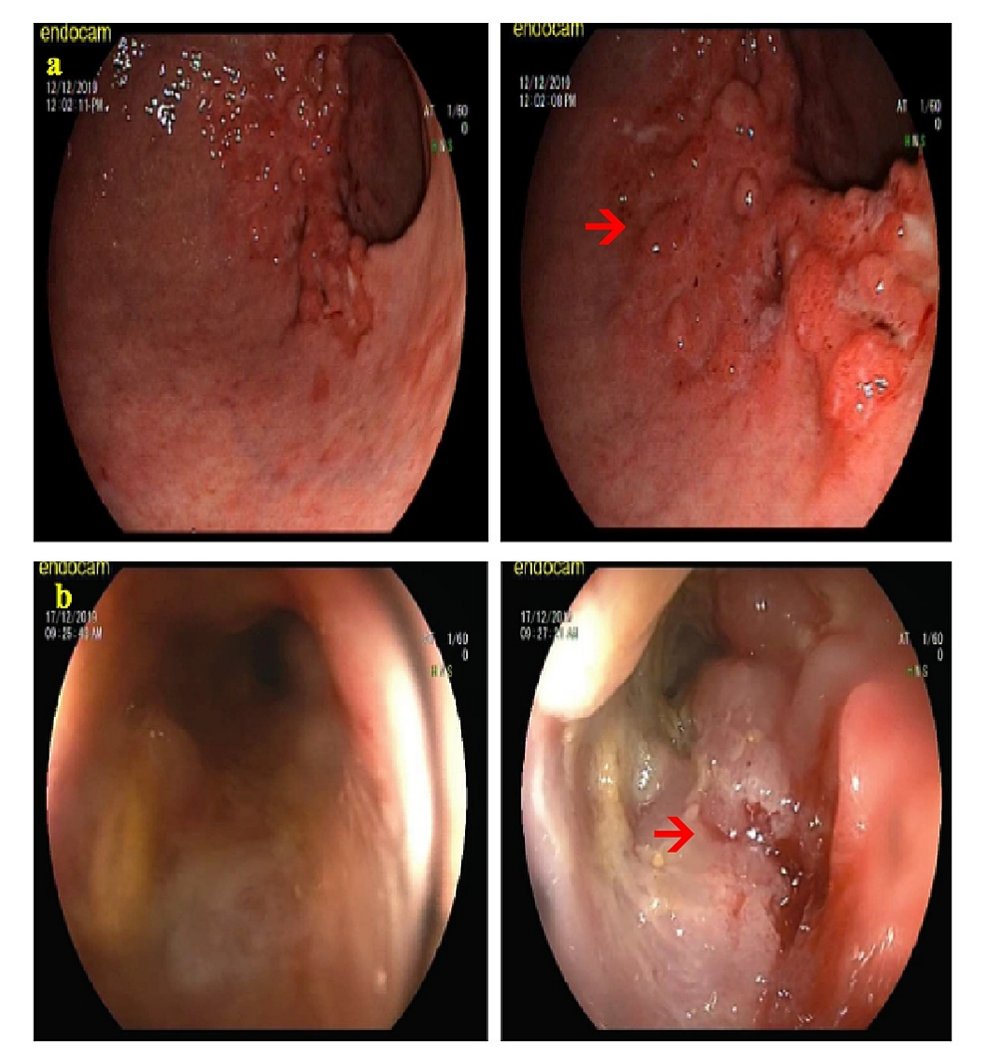
Preoperative endoscopic findings. (a) Polypoid lesions in the gastric antrum in esophagogastroduodenoscopy. (b) Colonoscopic view of an ulcerovegetan mass that completely surrounding the colon lumen, approximately 30 cm from the anal verge.

A 63-year-old Turkish male patient applied to the general surgery outpatient polyclinic with complaints of abdominal pain and constipation ongoing for nearly one year but increased in the past two months. His medical history revealed that the patient received medical treatment due to hypertension and anxiety disorder. He had no history of smoking or alcohol consumption. There was no history of cancer in the patient's first-degree relatives. His surgical history revealed an appendectomy nearly thirty years ago. The patient's body mass index (BMI) was 32 kg/m^2^ and physical examination showed a McBurney's incision scar. Esophagogastroduodenoscopy revealed polypoid lesions in the antrum, the largest measuring 1 cm in diameter, and biopsies were obtained for histopathological examination. Colonoscopy revealed an ulcerovegetan mass, approximately 30 cm from the anal verge, completely surrounding the colon lumen, and multiple biopsies were obtained for histopathological examination (Figure 1).

**Figure 2:**
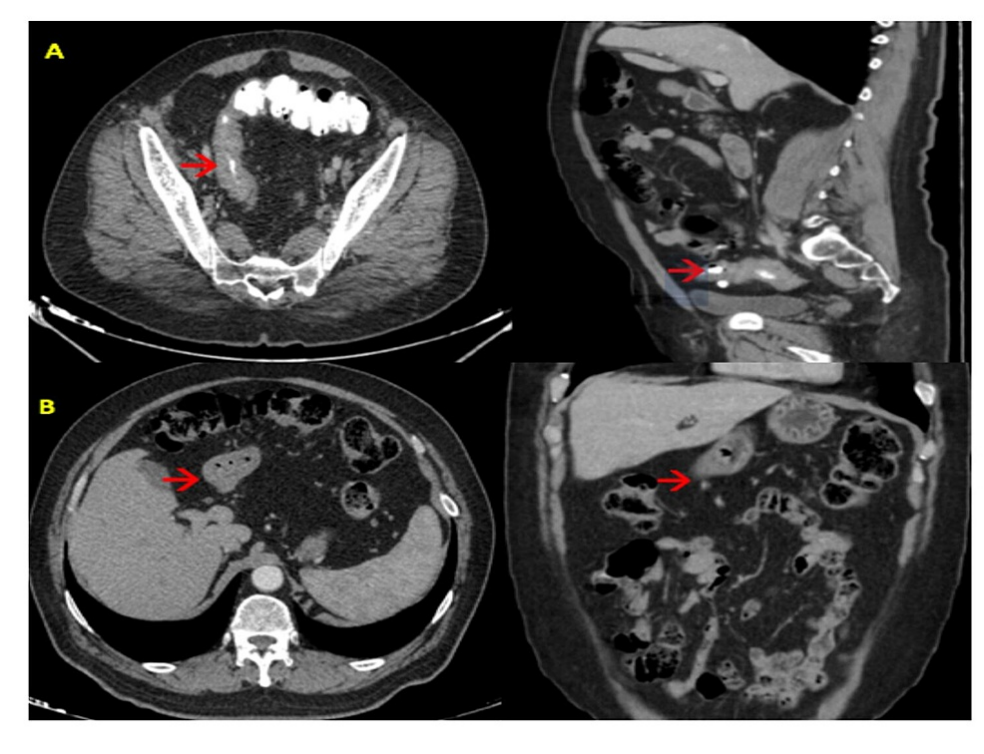
Preoperative CT findings. (A) Coronal and axial CT showed increased wall thickening of the sigmoid colon (red arrow). (B) Coronal and axial CT showed increased wall thickening of the gastric antrum (red arrow).

Histopathological examination results were reported as well-differentiated adenocarcinoma for the stomach and moderately differentiated adenocarcinoma for the colon. Abdominal and thoracic tomography (CT) for staging showed increased wall thickening of the sigmoid colon and gastric antrum (Figure 2).

No distant organ metastasis or pathologic lymph node was detected. The clinical staging was T2N0M0 - Stage I (American Joint Committee on Cancer (AJCC) 8th Edition) for the gastric cancer and T3N0M0 - Stage IIA (AJCC 8th Edition) for the colon cancer. Carcinoembryonic antigen (CEA) was 0.819 ng / mL, and cancer antigen 19-9 (CA 19-9) was 37 U / mL preoperatively. Based on these findings, a decision was made to perform simultaneous total laparoscopic distal gastrectomy (Roux-en-Y anastomosis and D2 lymph node dissection) + anterior resection. Both procedures were performed by the same surgeon (B.G.). The operating surgeon had experience in more than 50 open cases and in 30 laparoscopic cases for gastric cancer as well as more than 60 open cases and more than 80 laparoscopic cases for colorectal cancer.

Surgical procedures

**Figure 3:**
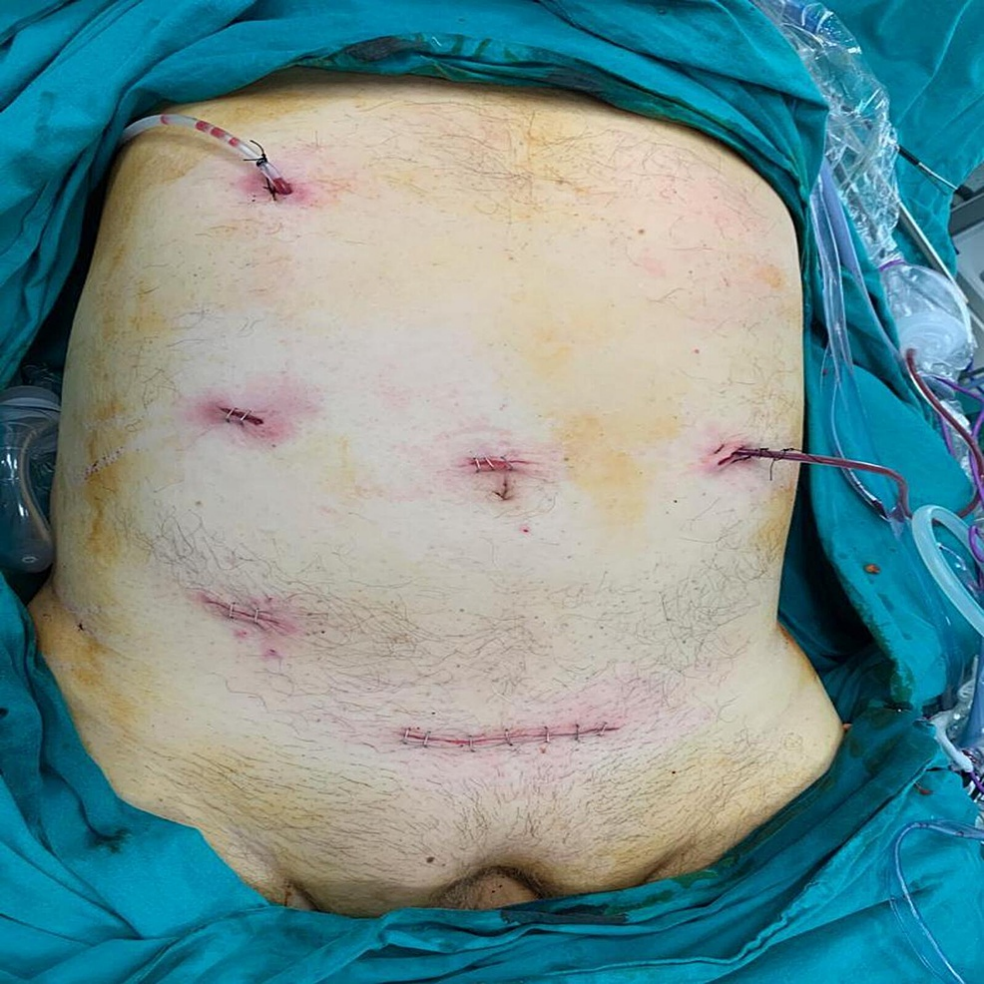
: Postoperative trocar and drain locations.

Under general anesthesia, the patient was given supine position with legs closed and the surgeon stood on the patient's right side. First the gastric operation, then the sigmoid colon surgery was planned. After creating pneumoperitoneum with a Veress needle, the pressure was adjusted as 10-12 mmHg using carbon dioxide gas. A 10-mm trocar was inserted through the supraumbilical region for a 30-degree-angled camera port. The exploration did not reveal any intra-abdominal ascites and/or liver metastasis and/or peritoneal implant. A total of four surgical trocars were used except the camera port: a 5-mm trocar along the umbilicus and right midclavicular line (shared trocar), a 5-mm trocar through the right midclavicular and subcostal area (for the stomach), a 12-mm trocar along the umbilicus and left midclavicular line (shared trocar) and a 12-mm trocar through the right lower quadrant (for the colon) (Figure 3).

**Figure 4:**
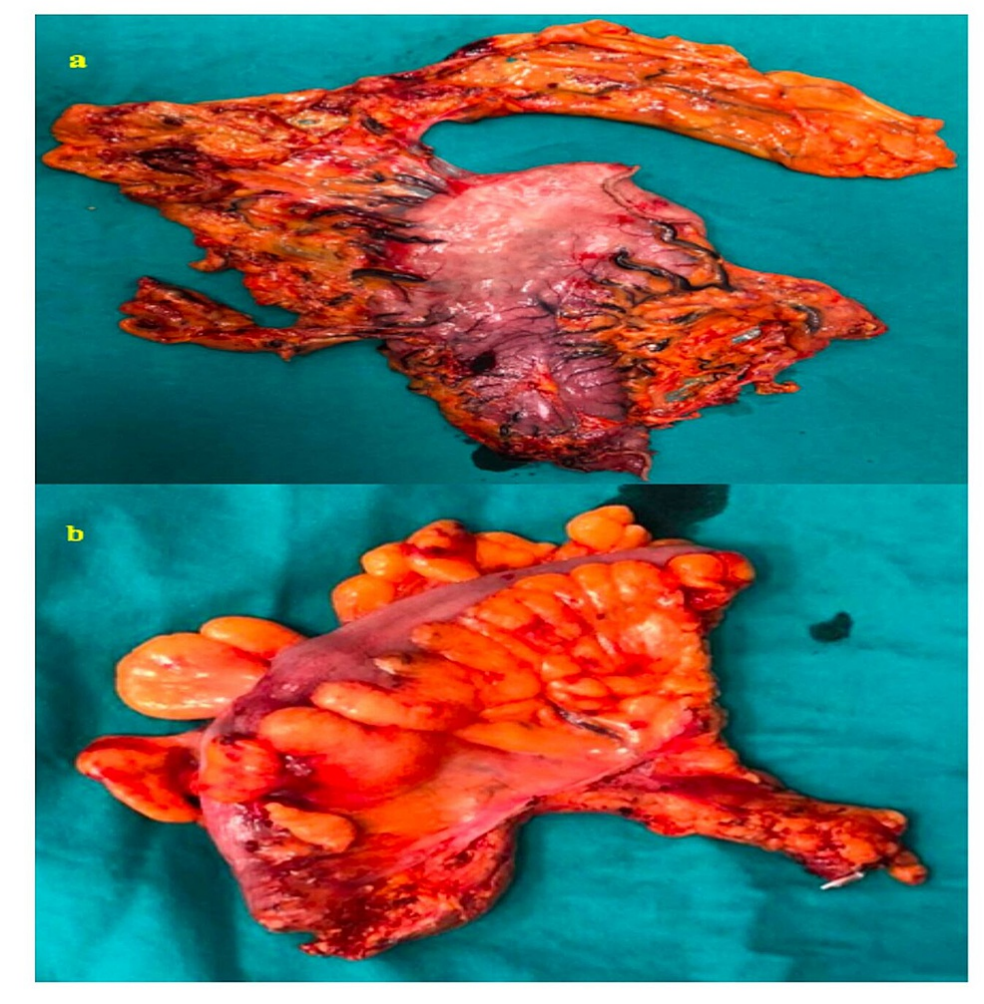
Postoperative gastric (a) and sigmoid colon (b) pieces.

Instead of using an additional port for liver retraction, both ends of a gauze opened longitudinally were tied with silk suture and inserted into the abdomen. Then, these sutures were held and suspended with a needle inserted into the abdomen through the xiphoid process. The patient was given reverse Trendelenburg position (30 degrees). The gastrocolic ligament was opened from the hepatic flexure towards the splenic flexure and the stomach was mobilized by ligating the gastroepiploic vessels. After the stomach was mobilized, the duodenum was cut and sutured 2 cm distal to the pylorus using a 45-mm linear stapler (Endo GIA™ Articulating Reload with Tri-Staple ™ Technology, Covidien™). After the right and left gastric vessels were ligated with a laparoscopic clip (Weck® Hem-O-Lok® Polymer Ligation, Teleflex Medical) and cut, two-thirds of the stomach was resected approximately 5 cm above the lesion using two 60-mm linear staplers, placed into a plastic bag and sealed. During the reconstruction phase, the jejunum was divided with a 45-mm linear stapler 20 cm from the ligament of Treitz. Distal jejunum was pulled up antecolically and side-to-side gastrojejunostomy anastomosis was performed from the posterior aspect of the gastric remnant towards the greater curvature using a 60-mm linear stapler. The stapler opening was closed with continuous suture (V-Loc™, Medtronic™). Side-to-side isoperistaltic jejunojejunostomy anastomosis was performed 40 cm distal to the gastrojejunostomy anastomosis with a 60-mm linear stapler. The stapler opening was closed with continuous suture (V-Loc™, Medtronic™). This phase of the surgery took 185 min. and estimated blood loss was 100 mL. Then, the patient was given Trendelenburg position (45 degrees) and the colon phase was started. Complete mesocolic excision was planned with a medial-to-lateral approach. As previously described [7], the surgeon took an atraumatic grasper in his left hand and a pair of scissors in his right hand and started the dissection after the sigmoid mesocolon was suspended. The visceral peritoneum was opened at the sacral promontorium level. The inferior mesenteric artery was ligated and cut at its aortic origin (high ligation) using a laparoscopic clip preserving the para-aortic nerves. Subsequently, the inferior mesenteric vein was ligated and cut below the pancreatic margin using a similar laparoscopic clip. Left side of the abdominal wall was accessed through a sharp medial-to-lateral dissection preserving the ureters. In the lateral aspect, the colon's suspensory ligaments were cut. In addition to the gastrocolic ligaments which were already cut during the gastric phase, the remaining splenocolic ligament was also cut and completely mobilized to the splenic flexure. Then, the proximal rectum was prepared and distal resection was performed intracorporeally with a 45-mm linear stapler. The abdomen was entered through a 5-cm Pfannenstiel incision. Using a wound protector cover (Alexis® Wound Protector/Retractor), firstly the gastric piece was removed from the abdomen. Subsequently, the colon was removed from the abdomen and proximal resection of the colon was performed at the distal descending colon (Figure 4).

**Figure 5:**
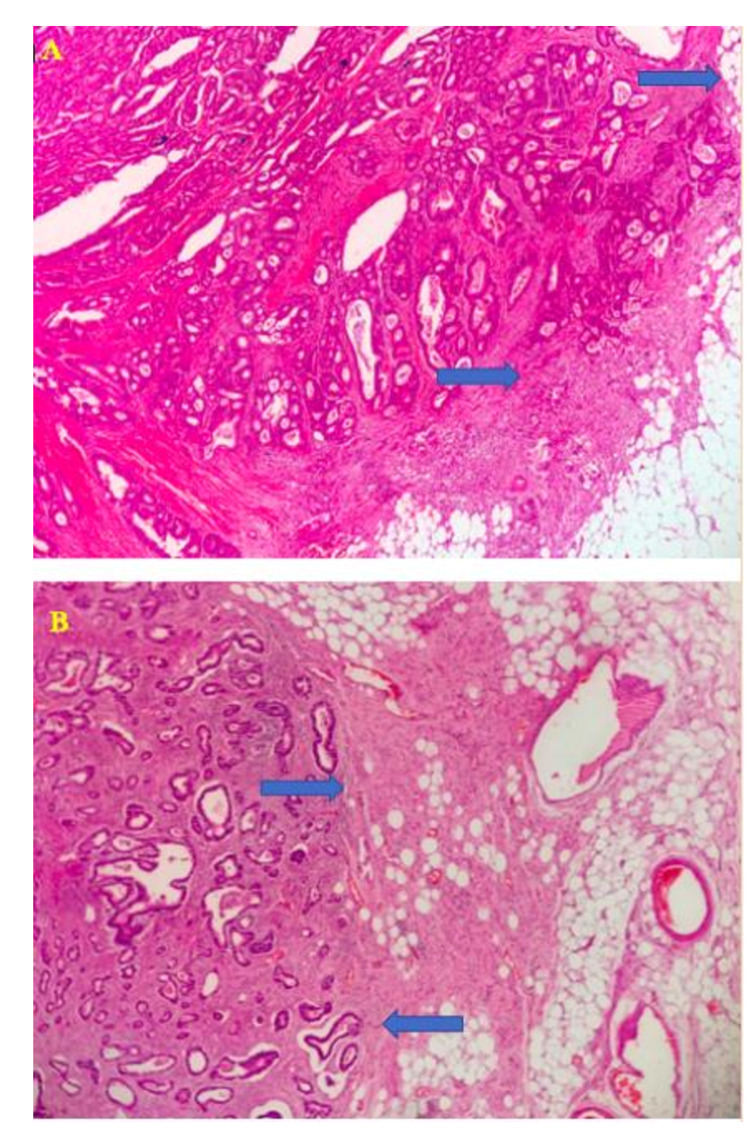
Histopathology evaluation. (A) Histopathological appearance of well-differentiated adenocarcinoma of the colon. Arrows mark the area of serosal invasion (H&E x40). (B) Histopathological appearance of moderately-differentiated adenocarcinoma of the stomach. Arrows mark the area of serosal invasion (H&E x40). H&E: hematoxylin and eosin.

After the anvil of a 31-mm circular stapler (Covidien® 31 mm - 4.8 mm) was placed at the descending colon with a purse-string suture, the pneumoperitoneum was recreated. Finally, the circular stapler device was placed through the anal route and end-to-end colorectal anastomosis was performed intracorporeally with the double-stapling technique. A total of two silicone aspiration drains were inserted, one to drain the area from the right subcostal trocar site to the duodenal stump and the posterior part of the gastrojejunostomy anastomosis and one to drain the area from the left quadrant trocar site to the posterior part of the colorectal anastomosis (Figure 3). This phase of the surgery took 125 min and the estimated blood loss was 75 mL. Thus, the procedure was completed with a total operation time of 310 min and the total estimated blood loss of 175 mL. Liquid food was started for the patient who had gas exit on postoperative Day 2 and solid food the next day. The patient's drains were removed and he was discharged without any complication on Day 5. Upon detecting minimal free air and fluid around the duodenal stump in the abdominal CT, the patient underwent an emergency operation considering possible duodenal stump leakage (DSL). The exploration performed with a midline incision above the umbilicus revealed duodenal stump leakage of 0.5 cm, while no widespread peritonitis was detected. Duodenorrhaphy with omentoplasty was performed. The patient stayed in the intensive care unit for 1 day postoperatively and was discharged on Day 20 without any complications. The pathologic examination result was reported as Grade I well-differentiated adenocarcinoma (pT3N0M0 - Stage IIA, AJCC 8th Edition) without lymph node metastasis (0/31) for the gastric cancer and Grade I moderately differentiated adenocarcinoma (pT3N0M0 - Stage IIA, AJCC 8th Edition) without lymph node metastasis (0/29) for the sigmoid colon cancer. The tumoral growths observed in the histopathological sections of both resection materials start from the mucosa and spread towards the serosa. In addition, due to the absence of tumor metastasis in the lymph nodes obtained from both resection materials, tumoral lesions observed in both organs were considered as separate primary tumors (Figure 5). The patient was administered postoperative adjuvant chemotherapy and remains under follow-up at 21 months without any recurrence. Written informed consent form was obtained from the patient before the procedure. The procedure was conducted in accordance with the committee's ethical standards (institutional and national) responsible for human experimentation and the 1964 Helsinki Declaration and its later versions.

## Discussion

Simultaneous open surgery for gastric and colon cancer usually requires a wide laparotomy incision. Minimally invasive surgery has been used commonly for both cancers for a long while. Moreover, simultaneous laparoscopic combined resections have started to be performed as a result of the advancements in minimally invasive surgical techniques [5,6,8,9]. Although no consensus has been reached on the organ to start with due to the limited number of reported cases, first the gastric, then the colon operations have been typically performed [5,8]. We started with the gastric operation as well due to a few reasons. The first reason was related to the principles of oncology. The preoperative staging demonstrated that the gastric cancer had an earlier stage. We planned to proceed with open surgery in terms of the principles of oncology considering the possible adjacent organ invasion in advanced-stage colon tumors, particularly T4 tumors. In such a scenario, if we had started with the colon operation, we would have to widen the midline incision during the gastric phase. The second reason was related to our plan to remove both pieces from the suprapubic region through a transverse incision (Pfannenstiel incision). For the colorectal anastomosis, the purse suture was placed at the descending colon through this incision. If we had started from the colon, any unfavorable condition requiring open surgery during the gastric phase would necessitate a midline incision. In such a case, both a midline incision and a suprapubic transverse incision would have been created.

Ojima et al. have reported six patients who underwent laparoscopic-assisted gastric and colorectal resection. Their mean operation time was 442 min. and they used 7-9 ports [6]. In our study, we performed the surgery successfully with a shorter operation time (310 min.) and used a smaller number of ports (5 trocars). The technique we used for liver retraction reduced the requirement for additional trocars.

The incidence of DSL is higher in laparoscopic surgery compared to open surgery [10]. In a study from Korea reporting eight cases undergoing simultaneous laparoscopic-assisted gastric and colon resection, biliary leakage was detected in one case, while no details were provided regarding the diagnosis or treatment [9]. A recent study demonstrated the non-reinforcement of the duodenal stump as the most significant risk factor for post-laparoscopic gastrectomy DSL [11]. Our patient was reoperated due to DSL four days after discharge (on postoperative Day 9). A reason for this may be the fact that we did not reinforce the duodenal stump. Besides this known reason, we observed during the exploration that the small intestines had entered below the mobilized mesocolon and were dilated. This may have caused increased intraluminal pressure in the small intestine trapped below the colon, leading to opening of the duodenal stump. During his previous laparoscopic left colon operations, the surgeon had not closed the opening occurring at the mesocolon. However, closing the mesocolon opening may have been considered in this unusual case.

Another issue to discuss is the site selected for specimen removal. In the majority of the above-mentioned studies, the specimen was removed through the midline mini-laparotomy incision. Pfannenstiel incision is the type of incision providing the lowest risk for incisional hernia and wound site infection after laparoscopic colorectal operations [12]. Another advantage is the better cosmetic appearance of this incision. This is another significant feature of our study distinguishing it from other similar studies.

## Conclusions

In this study, we presented a case who underwent simultaneous total laparoscopic curative surgery for synchronous gastric and sigmoid colon cancer. The number of simultaneous operations for gastric and colon cancers has started to increase due to the advancements in diagnostic tests and minimally invasive surgical techniques. However, there are still technical issues to be discussed. A more standard approach may be recommended in the future with large case series.
